# Proteomic identification of *Acanthamoeba castellanii* excretory–secretory proteins associated with *Klebsiella pneumoniae* capsule enlargement: a candidate role for C-type lysozyme

**DOI:** 10.1051/parasite/2026057

**Published:** 2026-10-02

**Authors:** Chun-Hsien Chen, Jian-Ming Huang, Wei-Chen Lin, Chih-Ming Tsai, Hsin-Yu Hsieh, Yu-Jen Wang

**Affiliations:** 1 Graduate Institute of Biomedical Sciences, College of Medicine, Chang Gung University Taoyuan Taiwan; 2 Department of Parasitology, College of Medicine, Chang Gung University Taoyuan City Guishan Dist. Taiwan; 3 School of Medicine, National Tsing Hua University Hsinchu Taiwan; 4 Institute of Molecular and Cellular Biology, National Tsing Hua University Hsinchu Taiwan; 5 Department of Parasitology, College of Medicine, National Cheng Kung University Tainan Taiwan; 6 Institute of Basic Medical Sciences, College of Medicine, National Cheng Kung University Tainan Taiwan; 7 Department of Parasitology, College of Medicine, National Cheng Kung University Tainan Taiwan; 8 Department of Nursing, Feng Yuan Hospital, Ministry of Health and Welfare Taichung Taiwan; 9 Department of Parasitology, School of Medicine, China Medical University Taichung Taiwan; 10 Graduate Institute of Biomedical Sciences, China Medical University Taichung Taiwan; 11 Departments of Laboratory Medicine and Internal Medicine, China Medical University Hospital Taichung Taiwan

**Keywords:** *Acanthamoeba castellanii*, *Klebsiella pneumoniae*, Secretome, Label-free proteomics, Rcs two-component system, Lysozyme

## Abstract

*Background*: *Acanthamoeba castellanii* and *Klebsiella pneumoniae* are environmental commensals that co-exist in aquatic habitats. Our previous studies demonstrated that *A. castellanii* genotype T4 (AcT4) induces capsule enlargement and hypermucoviscosity in *K. pneumoniae* through small excretory–secretory proteins (ESPs; <10 kDa) that activate the Rcs two-component system, upregulating capsule biosynthesis genes independently of virulence plasmids. However, the molecular identity of these active ESPs remained undefined. *Results*: To identify candidate proteins, we performed label-free quantitative proteomics (LC-MS/MS) on conditioned medium from AcT4 co-cultured with *K. pneumoniae* (Ac/Kp) *versus* AcT4 alone (Ac only; *n* = 3 per group). A total of 391 *A. castellanii* proteins were identified; seven showed significantly increased extracellular abundance in Ac/Kp medium (log_2_ ratio ≥ 1, *p* < 0.05), and 98 showed decreased abundance. Functional enrichment analysis revealed that increased-abundance proteins were enriched in transport, signaling/regulation, redox activity, ion/metal binding, and chaperone/stress response categories. Among these, a C-type lysozyme/α-lactalbumin superfamily protein (UniProt L8GZI4; 14.7 kDa; log_2_ ratio = 5.19) was identified as a priority candidate, as lysozyme-mediated peptidoglycan hydrolysis generates muropeptides—known activators of the Rcs phosphorelay. *Conclusions*: This comparative secretome analysis provides the first proteomic catalogue of AcT4 extracellular proteins during co-culture with *K. pneumoniae* and identifies C-type lysozyme as a biologically plausible candidate mediating Rcs-dependent capsule induction. These findings lay the groundwork for future validation of AcT4-derived ESPs in protozoa–bacteria interactions and their potential role in the environmental emergence of hypervirulent *K. pneumoniae*.

## Abstract (fr)

*Contexte* : *Acanthamoeba castellanii* et *Klebsiella pneumoniae* sont des organismes commensaux présents dans l'environnement, qui coexistent dans les habitats aquatiques. Nos études précédentes ont démontré qu'*A. castellanii* de génotype T4 (AcT4) induit une augmentation de la taille de la capsule et une hypermucoviscosité chez K*. pneumoniae* par l'intermédiaire de petites protéines excrétées/sécrétées (PES, < 10 kDa) ; celles-ci activent le système à deux composants Rcs, entraînant une régulation à la hausse des gènes de biosynthèse de la capsule indépendamment des plasmides de virulence. Toutefois, l'identité moléculaire de ces PES actives restait à déterminer. *Résultats* : Afin d'identifier des protéines candidates, nous avons réalisé une analyse protéomique quantitative sans marquage (LC-MS/MS) sur le milieu conditionné issu de la coculture d'AcT4 avec *K. pneumoniae* (Ac/Kp) par rapport à celui d'AcT4 seul (Ac seul ; *n* = 3 par groupe). Au total, 391 protéines d'*A. castellanii* ont été identifiées ; sept présentaient une abondance extracellulaire significativement accrue dans le milieu Ac/Kp (rapport log_2_ ≥ 1, p < 0,05), tandis que 98 présentaient une abondance réduite. L'analyse d'enrichissement fonctionnel a révélé que les protéines dont l'abondance était accrue appartenaient aux catégories suivantes : transport, signalisation/régulation, activité redox, liaison aux ions/métaux, et chaperonnes/réponse au stress. Parmi elles, une protéine de la superfamille des lysozymes de type C/α-lactalbumines (UniProt L8GZI4 ; 14,7 kDa ; rapport log_2_ = 5,19) a été identifiée comme candidate prioritaire, car l'hydrolyse du peptidoglycane médiée par le lysozyme génère des muropeptides, connus pour activer le système de phosphorelais Rcs. *Conclusions* : Cette analyse comparative du sécrétome fournit le premier catalogue protéomique des protéines extracellulaires d'AcT4 lors de la coculture avec *K. pneumoniae* et identifie le lysozyme de type C comme un candidat biologiquement plausible pour médier l'induction de la capsule via le système Rcs. Ces résultats jettent les bases d'une validation ultérieure des PES dérivés d'AcT4 dans le cadre des interactions protozoaires-bactéries et de leur rôle potentiel dans l'émergence environnementale de *K. pneumoniae* hypervirulentes.

## Introduction

*Klebsiella pneumoniae* is a Gram-negative opportunistic pathogen responsible for a wide spectrum of community-acquired and nosocomial infections, including pneumonia, urinary tract infection, liver abscess, and septicaemia [20]. Of particular concern is the emergence of hypervirulent *K. pneumoniae* (hvKp), which is characterised by its hypermucoviscous capsule, enhanced resistance to host immune clearance, and capacity to cause invasive infections in immunocompetent individuals [24]. While the genetic basis of hypervirulence has been extensively studied, the environmental conditions and microbial interactions that may drive the transformation of classical *K. pneumoniae* (cKp) into hvKp in natural habitats remain incompletely understood. *Acanthamoeba* spp. are free-living amoebae ubiquitous in soil, freshwater, and man-made water systems, including hospital water networks [26]. As voracious environmental phagocytes, *Acanthamoeba* spp. exert considerable selective pressure on co-occurring microorganisms and are recognised as important drivers of bacterial virulence evolution in aquatic environments [23]. Both *K. pneumoniae* and *Acanthamoeba* spp. co-exist in shared environmental niches; their co-occurrence in natural freshwater systems has been directly confirmed by field surveillance in urban river systems in Taiwan, where both organisms were detected at overlapping sampling sites [8]. These observations suggest that their interaction in natural settings may have broader implications for the emergence of pathogenic bacterial strains.

Our previous work demonstrated that *Acanthamoeba castellanii* genotype T4 (AcT4) can induce capsule enlargement and hypermucoviscosity in *K. pneumoniae* during co-culture, and that this effect is dependent on the number of amoeba cells rather than the density of bacteria [11]. Importantly, the induced hypermucoviscous phenotype was heritable across subsequent bacterial generations in the absence of *Acanthamoeba*, and was observed across multiple clinical and environmental *K. pneumoniae* serotypes. In a follow-up study, we showed that AcT4-conditioned medium enhanced *K. pneumoniae* cytotoxicity and serum resistance without altering antibiotic susceptibility, and that the molecular mechanism involves activation of the Rcs two-component system [4]. The Rcs phosphorelay is a well-characterised regulatory pathway in Enterobacteriaceae that responds to cell envelope stress and promotes capsular polysaccharide biosynthesis by upregulating the *cps* locus genes, including *rcsB*, *galF*, and *wzi* [7]. Fractionation of AcT4 excretory–secretory proteins (ESPs) further demonstrated that molecules below 10 kDa constitute the primary active fraction responsible for capsule induction. However, the molecular identity of these small ESPs and the mechanism by which they activate the Rcs system in *K. pneumoniae* remained undefined.

Characterisation of the *Acanthamoeba* secretome has been greatly facilitated by advances in mass spectrometry-based proteomics. A previous study identified 130 proteins in *A. castellanii* exosome-like vesicles by LC-MS/MS [15], demonstrating that the amoeba extracellular proteome is diverse and functionally complex. To identify the candidate AcT4 proteins responsible for *K. pneumoniae* capsule induction, we performed label-free quantitative proteomics on conditioned medium collected from AcT4 co-cultured with *K. pneumoniae* compared with AcT4 cultured alone. This comparative secretome analysis aimed to identify *A. castellanii* proteins whose extracellular abundance is specifically altered during bacterial co-culture, and to prioritise candidate ESPs whose molecular weight, secretory status, and biochemical activity are mechanistically consistent with Rcs phosphorelay activation in *K. pneumoniae*.

## Materials and methods

### Ethics statement

The use of the clinical *Klebsiella pneumoniae* isolate K-81, originally recovered from the corneal surface of a hospitalised patient, was approved by the Institutional Review Board (IRB) of National Cheng Kung University Hospital, Tainan, Taiwan (Protocol No. B-ER-109-108; approval date: 8 June 2020). No additional ethical approval was required for the present study.

### Cultivation of *Acanthamoeba castellanii*

The genotype T4 strain of *A. castellanii* (ATCC 30010; Neff strain) was obtained from the American Type Culture Collection (ATCC, Manassas, VA, USA) and maintained as described previously (Chuang *et al.*, 2025). Briefly, trophozoites were cultured at 28 °C in proteose peptone–yeast extract–glucose (PYG) medium supplemented with 0.98 g/L MgSO_4_, 0.34 g/L KH_2_PO_4_, 0.188 g/L Na_2_HPO_4_·7H_2_O, and 0.02 g/L Fe(NH_4_)_2_(SO_4_)_2_·6H_2_O (pH 6.5). Before each experiment, amoeba cells were harvested by centrifugation, washed three times in Page’s modified Neff’s amoeba saline (PAS; 1.2 g/L NaCl, 0.04 g/L MgSO_4_·7H_2_O, 0.03 g/L CaCl_2_, 1.42 g/L Na_2_HPO_4_, 1.36 g/L KH_2_PO_4_), and resuspended at the desired density.

### Cultivation of *Klebsiella pneumoniae*

The *K. pneumoniae* isolate K-81 (serotype K81), previously recovered from the corneal surface of a hospitalised patient and characterised in our earlier studies [11], was routinely cultured on Luria-Bertani (LB) agar or in LB broth at 35 °C. For co-culture experiments, an overnight culture of K-81 was diluted in fresh LB broth and grown to the exponential phase; bacteria were then harvested by centrifugation at 3,000× *g* for 5 min and resuspended in PAS buffer at the required optical density.

### Co-culture and conditioned medium collection

*Acanthamoeba castellanii* trophozoites were washed and resuspended in PAS buffer at a density of 6 × 10^6^ cells/mL. *Klebsiella pneumoniae* K-81 cells were prepared at an OD_600_ of 0.2 in PAS buffer to obtain a concentration of 1 × 10^8^ CFU/mL. Equal volumes of the two suspensions were mixed and co-cultured in a petri dish at 28 °C for 24 h (Ac/Kp group). A parallel control consisting of *A. castellanii* in PAS buffer without bacteria was maintained under identical conditions (Ac only group). Three independent biological replicates were prepared for each group. After incubation, the co-culture suspensions were centrifuged at 2,000× *g* for 10 min at 4 °C to remove cells and debris, and the resulting conditioned medium (supernatant) was collected. Each conditioned medium sample was further clarified by filtration through a 0.22 μm membrane and stored at −80 °C until proteomics analysis.

### SDS-PAGE analysis of conditioned medium proteins

Equal volumes of each conditioned medium sample were resolved on a 10% sodium dodecyl sulfate–polyacrylamide gel and visualised by Coomassie Brilliant Blue staining (Fig. 2).

### In-solution tryptic digestion

Sample preparation and tryptic digestion were performed by Biotool Co., Ltd. (Taipei, Taiwan; Service No. QT24050019). Conditioned medium proteins were reconstituted in 50 mM ammonium bicarbonate (Sigma-Aldrich). Disulfide bonds were reduced with 10 mM dithiothreitol (Merck) at 56 °C for 45 min, followed by cysteine alkylation with 55 mM iodoacetamide (Sigma-Aldrich) at 25 °C for 30 min in the dark. Samples were digested overnight at 37 °C with sequencing-grade modified porcine trypsin (Promega, Madison, WI, USA) at a protein-to-enzyme ratio of 20:1. The resulting peptides were desalted, dried by vacuum centrifugation, and stored at −80 °C until LC-MS/MS analysis.

### Liquid chromatography–tandem mass spectrometry

Dried peptides were reconstituted in HPLC buffer A (0.1% formic acid) and loaded onto a trap column (Zorbax 300SB-C18, 0.3 × 5 mm; Agilent Technologies, Santa Clara, CA, USA). Chromatographic separation was performed on a reversed-phase column (Waters BEH C18, 1.7 μm, 100 μm I.D. × 10 cm, 15 μm emitter tip) using a 70-min gradient of HPLC buffer B (99.9% acetonitrile/0.1% formic acid) at a flow rate of 0.3 μL/min. The LC system was coupled to an Orbitrap Elite ETD mass spectrometer (Thermo Fisher Scientific, Waltham, MA, USA), operated via Xcalibur 2.2 software. Full-scan spectra were acquired over a mass range of 400–2,000 Da at a resolving power of 120,000 at m/z 400, with internal calibration using the ion signal of protonated dodecamethylcyclohexasiloxane (m/z 536.165365) as a lock mass. Data-dependent acquisition selected the 20 most abundant precursor ions per full-scan cycle for CID fragmentation; dynamic exclusion was applied with a window of 40 s and a mass tolerance of 15 ppm. The electrospray voltage was set to 2.0 kV and the capillary temperature to 200 °C. Automatic gain control targets were set to 3 × 10^6^ ions (maximum 1,000 ms) for full-scan acquisition and 3,000 ions (maximum 200 ms) for MS/MS acquisition.

### Protein identification and database search

Raw MS data were processed using Proteome Discoverer software (version 2.3; Thermo Fisher Scientific). Peptide and protein identification was performed with the Mascot search engine (version 2.5; Matrix Science, London, UK) against the UniProt reference proteome of *A. castellanii* (strain ATCC 30010/Neff). To exclude potential cross-species false-positive matches or bacterial protein interference arising from co-culture conditions, potential contributions from *K. pneumoniae* were systematically ruled out during the analytical workflow, and identified peptides of the prioritized candidate were cross-referenced and validated against the *K. pneumoniae* reference proteome, confirming that no bacterial peptides were incorrectly assigned to *A. castellanii*. Search parameters included a precursor ion mass tolerance of 10 ppm and a CID fragment ion tolerance of 0.5 Da, with a maximum of two missed trypsin cleavage sites permitted. Oxidation of methionine and acetylation of protein N-termini were set as variable modifications; carbamidomethylation of cysteine was applied as a fixed modification. Peptide–spectrum matches (PSMs) were filtered at high confidence (false discovery rate ≤ 0.01, determined by the Target-Decoy PSM Validator algorithm) and rank 1. Proteins were accepted if identified by at least two unique peptides.

### Label-free quantification and statistical analysis

Label-free quantification (LFQ) was performed using normalised peptide abundances as calculated by Proteome Discoverer 2.3. All downstream statistical analyses and visualisations were performed in Python (version 3.12; Python Software Foundation). Missing abundance values were imputed with one-half of the minimum detected value within each sample (half-minimum imputation). Differential abundance between the Ac/Kp and Ac only groups was assessed by Welch’s two-sample *t*-test applied to log_2_-transformed abundances (*n* = 3 per group), implemented using the scipy.stats module (SciPy version 1.17.1). Proteins with |log_2_(abundance ratio)| ≥ 1 and *p* < 0.05 were classified as significantly differentially abundant. Principal component analysis (PCA) was performed on the log_2_-transformed, imputed abundance matrix using sklearn.decomposition.PCA (scikit-learn version 1.8.0). Pairwise Pearson correlation coefficients among biological replicates were calculated based on normalised protein abundances to evaluate quantitative reproducibility. Volcano plot, hierarchical clustering heatmap, and enrichment plots were generated using matplotlib (version 3.10.8) and numpy (version 2.4.4). Hierarchical clustering of the top differentially abundant proteins was performed using average linkage with the correlation distance metric, as implemented in scipy.cluster.hierarchy.

### Venn diagram analysis

Proteins detected with high confidence (‘High’ PSM identification status in ≥ 2 of 3 biological replicates) in at least one experimental condition were included in the Venn diagram analysis. The numbers of proteins unique to the Ac only condition, unique to the Ac/Kp condition, or detected in both conditions were determined using the vendor-provided analytical pipeline (Biotool Co., Ltd.) and visualised as a two-set Venn diagram (Fig. 1A).

**Figure 1:**
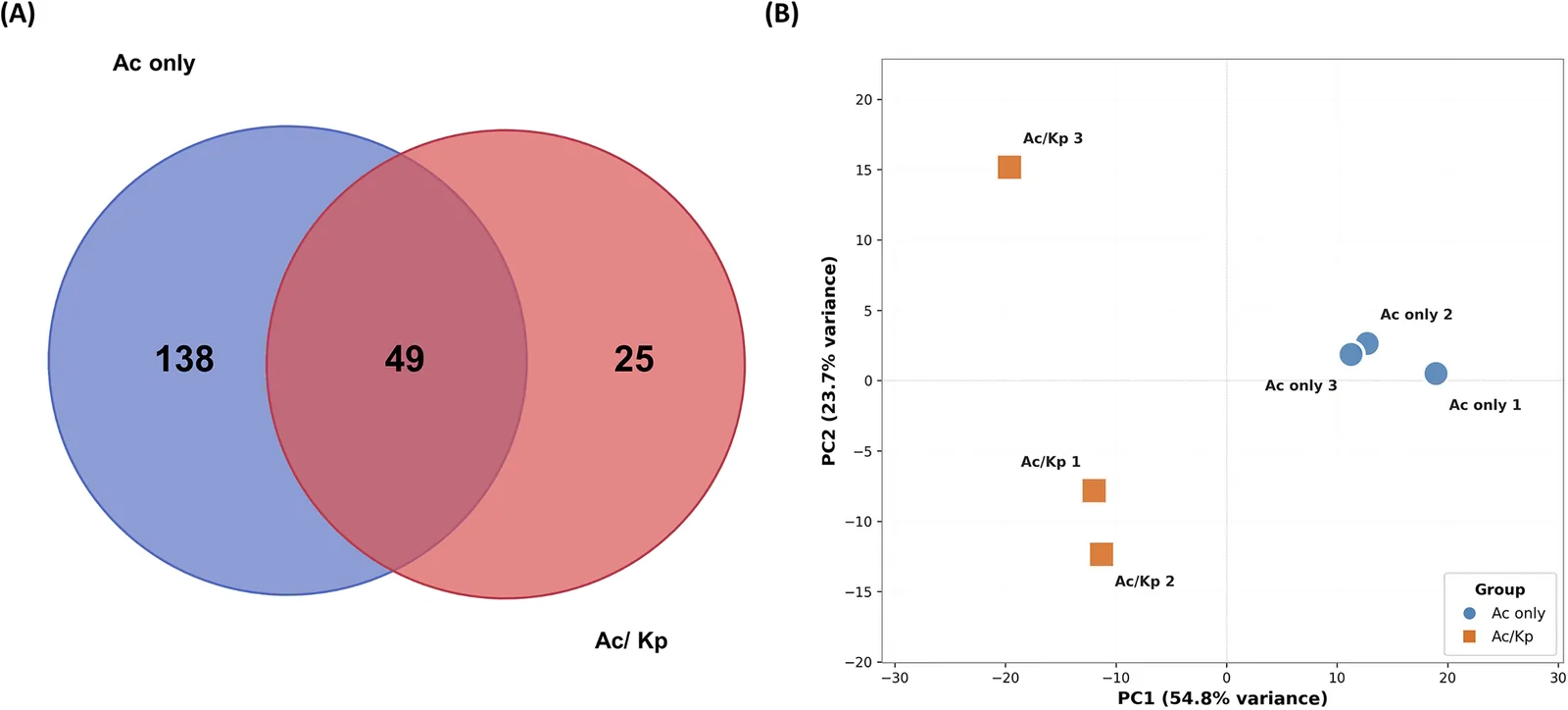
Sample quality control and overview of identified proteins in *Acanthamoeba castellanii* conditioned medium. (A) Venn diagram showing the distribution of high-confidence proteins (detected with “High” peptide-spectrum match confidence in ≥2 of 3 biological replicates) identified in conditioned medium from *A. castellanii* genotype T4 cultured alone (Ac only; left circle, blue) and co-cultured with *Klebsiella pneumoniae* K-81 (Ac/Kp; right circle, red). Numbers indicate proteins detected exclusively in each condition or shared between both conditions. (B) Principal component analysis (PCA) score plot of normalised protein abundance profiles from all six conditioned medium samples (Ac only: *n* = 3, circles; Ac/Kp: *n* = 3, squares). Each data point represents one biological replicate. The percentage of total variance explained by each principal component is shown on the corresponding axis.

### Functional enrichment analysis

Gene Ontology (GO) functional category enrichment and KEGG pathway enrichment analyses were performed on proteins with increased extracellular abundance in the Ac/Kp condition (log_2_ ratio > 0; detected in ≥ 2 of 3 Ac/Kp replicates; *n* = 28 proteins), against the background of all 391 identified proteins. Enrichment significance was determined by Fisher’s exact test (one-sided); categories and pathways with *p* < 0.05 were considered significantly enriched. GO functional category assignments were based on UniProt-annotated Gene Ontology terms; KEGG pathway assignments were based on keyword-level mapping of protein descriptions to KEGG pathway identifiers. Analyses were performed using the vendor-provided pipeline (Biotool Co., Ltd.).

## Results

### Sample quality control and reproducibility

To characterise the extracellular protein composition of *Acanthamoeba castellanii* during co-culture with *Klebsiella pneumoniae*, conditioned medium was collected from three independent biological replicates per condition (Ac only and Ac/Kp) and analysed by label-free quantitative proteomics. Equal volumes of conditioned medium were resolved by SDS-PAGE prior to LC-MS/MS analysis; Coomassie staining confirmed protein integrity across all six samples and revealed an overall reduction in total protein content in the Ac/Kp condition relative to Ac only (Fig. 2). PCA of the normalised protein abundance profiles demonstrated clear separation between the two groups along PC1, which accounted for 54.8% of the total variance, reflecting the prominent remodelling of the extracellular proteome elicited by bacterial encounter (Fig. 1B). PC2 accounted for a further 23.7% of total variance, with Ac/Kp replicate 3 displaying minor dispersion from replicates 1 and 2. Pairwise correlation analysis of normalised protein abundances confirmed robust intra-group reproducibility, with Pearson correlation coefficients between Ac/Kp replicate 3 and replicates 1 and 2 reaching 0.977 and 0.981, respectively (comparable to *r* = 0.949–0.988 among Ac only replicates), while inter-group correlations remained markedly low (*r* = 0.045–0.145). This indicates that the overall quantitative proteomic profiles were highly reproducible, and the dispersion along PC2 primarily reflects minor biological variability inherent to dynamic amoeba–bacteria co-cultures rather than technical inconsistency. Taken together, these results indicate that co-culture with *K. pneumoniae* induces reproducible and substantial remodelling of the *A. castellanii* extracellular proteome.

**Figure 2:**
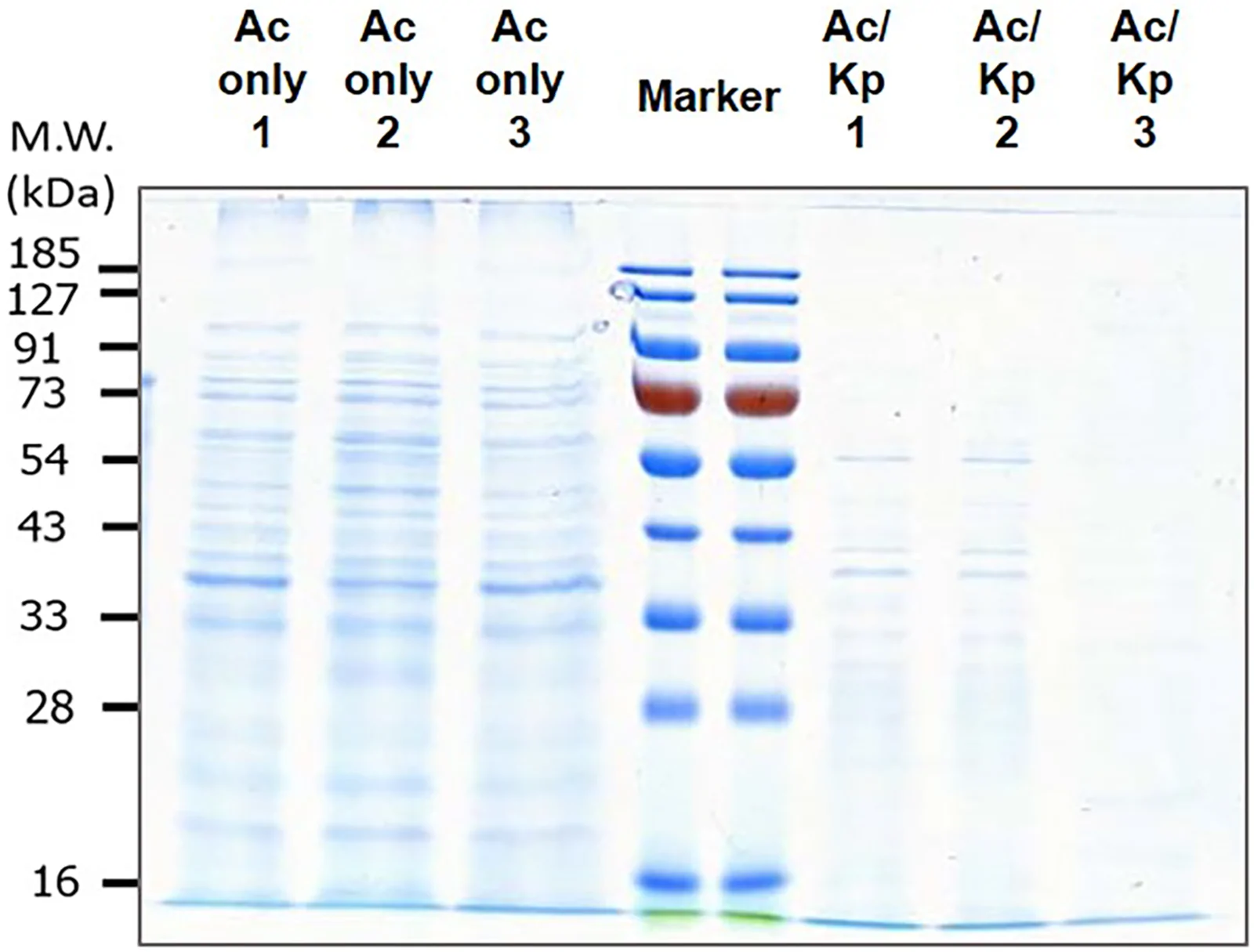
SDS-PAGE analysis of conditioned medium proteins. Equal volumes of conditioned medium from each biological replicate (Ac only 1–3 and Ac/Kp 1–3) were resolved on a 10% sodium dodecyl sulfate–polyacrylamide gel and visualised by Coomassie Brilliant Blue staining. Molecular weight marker positions (kDa) are indicated on the left. The gel confirms protein integrity across all six samples and reveals an overall reduction in total protein content in the Ac/Kp co-culture conditioned medium relative to the Ac only condition.

### Overview of identified proteins

A total of 391 *A. castellanii* proteins were identified across all six samples, applying a false discovery rate (FDR) of ≤ 0.01 and a minimum of two unique peptides per protein. Proteins were subsequently classified based on high-confidence detection (“High” status in ≥ 2 of 3 biological replicates) within each condition. Of the 391 identified proteins, 212 met this criterion in at least one condition. Venn diagram analysis revealed that 138 of these proteins were detected exclusively in the Ac only condition, 49 were detected in both conditions, and 25 were detected exclusively in the Ac/Kp co-culture condition (Fig. 1A). The presence of 25 proteins uniquely detected in the Ac/Kp condition, together with the loss of 138 proteins relative to Ac only, demonstrates that co-culture with *K. pneumoniae* substantially alters the composition of the *A. castellanii* extracellular proteome.

### Differential abundance analysis

Differential abundance analysis was performed on all 391 identified proteins using Welch’s two-sample *t*-test applied to log_2_-transformed, half-minimum-imputed normalised intensities (*n* = 3 per group). Proteins were classified as differentially abundant if they met the criteria of |log_2_(ratio)| ≥ 1 and *p* < 0.05. A total of 105 differentially abundant proteins (DAPs) were identified: seven proteins showed significantly increased extracellular abundance in the Ac/Kp co-culture conditioned medium, while 98 proteins showed significantly decreased abundance (Fig. 3). The remaining 286 proteins did not meet the significance thresholds and were considered unchanged. Hierarchical clustering of the top 20 proteins with increased abundance and the top 15 proteins with decreased abundance confirmed consistent group-wise differences across all replicates, with Ac/Kp samples exhibiting markedly higher or lower normalised abundances for the respective protein sets (Fig. 4).

**Figure 3:**
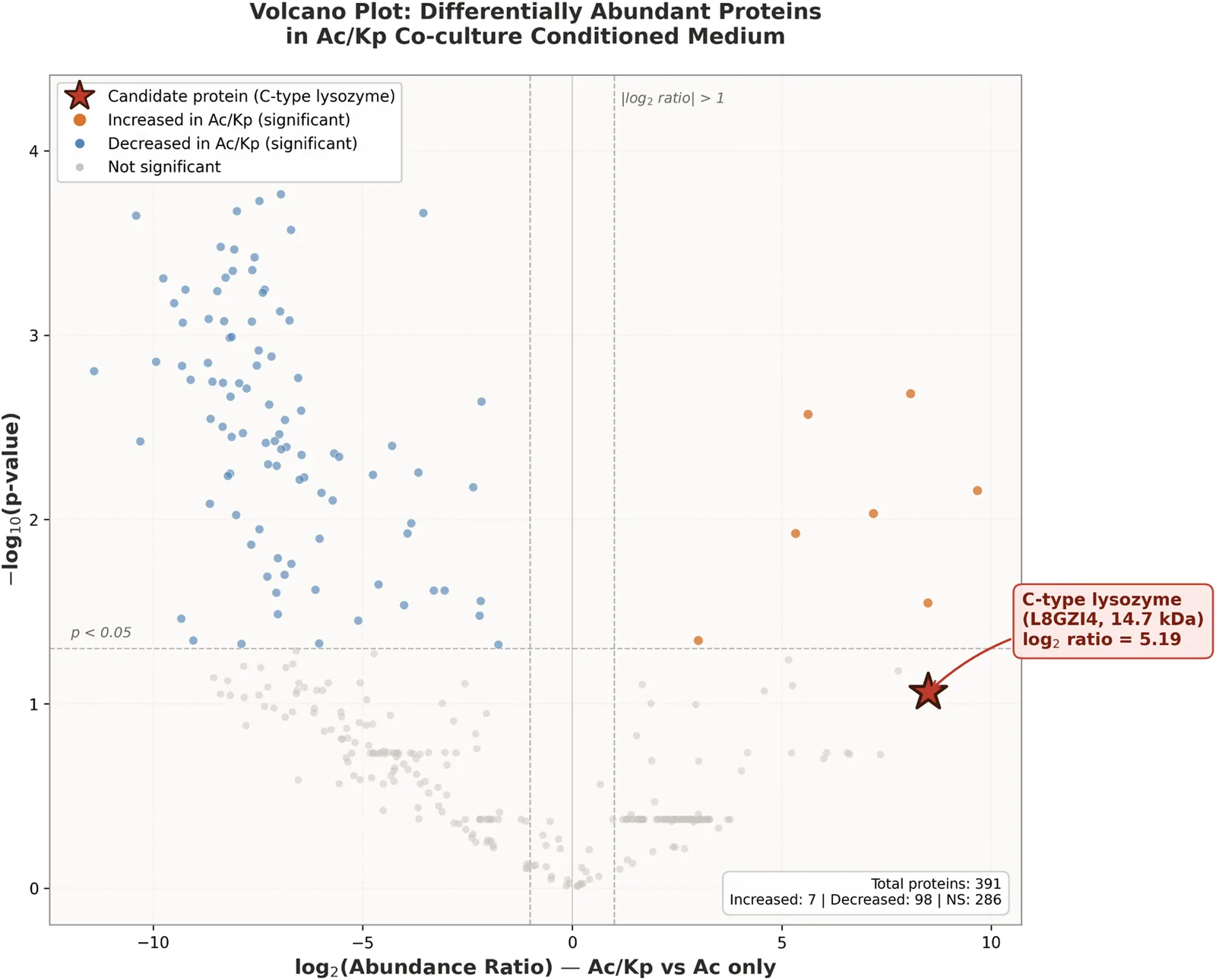
Volcano plot of differentially abundant proteins in Ac/Kp co-culture conditioned medium. Proteins identified across all samples (*n* = 391) are plotted according to their log_2_ (abundance ratio) (Ac/Kp *vs.* Ac only; *x*-axis) and negative log_10_-transformed Welch’s *t*-test *p*-value (*y*-axis). Proteins with |log_2_ ratio| ≥ 1 and *p* < 0.05 are considered significantly differentially abundant: those with increased abundance in Ac/Kp conditioned medium are shown in orange (*n* = 7); those with decreased abundance are shown in blue (*n* = 98); non-significant proteins are shown in grey (*n* = 286). The vertical dashed lines indicate |log_2_ ratio| = 1; the horizontal dashed line indicates *p* = 0.05. The priority candidate C-type lysozyme/α-lactalbumin superfamily protein (UniProt accession L8GZI4) is highlighted with a star symbol. The summary statistics (total proteins, number increased, decreased, and not significant) are indicated in the lower right corner of the plot.

**Figure 4:**
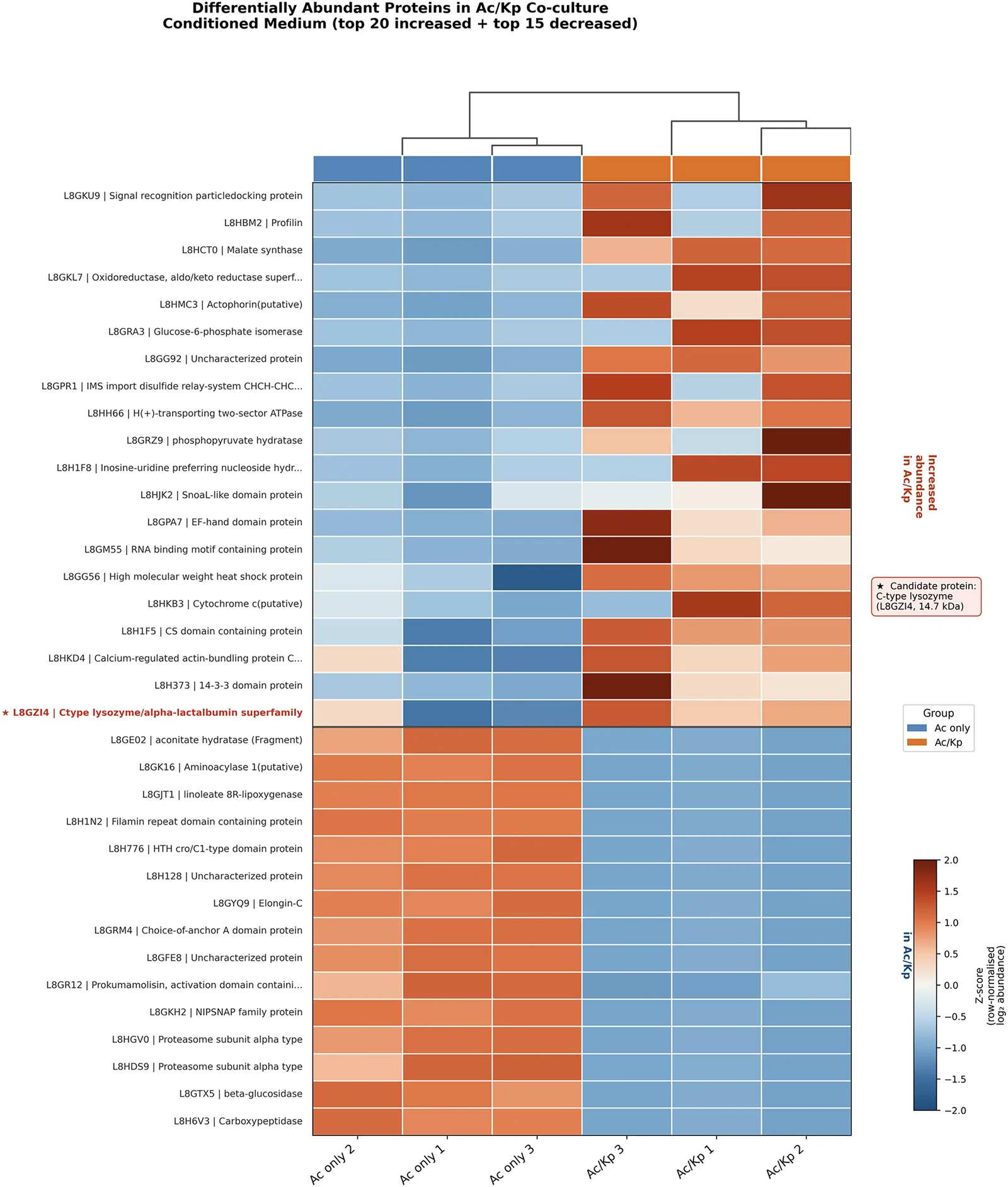
Hierarchical clustering heatmap of differentially abundant proteins. *Z*-score-normalised log_2_ protein abundances are shown for the top 20 proteins with the greatest increase in extracellular abundance in Ac/Kp conditioned medium (upper panel, warm colours) and the top 15 proteins with the greatest decrease (lower panel, cool colours). Columns represent individual biological replicates (Ac only 1–3; Ac/Kp 1–3) arranged by hierarchical clustering of samples (average linkage; Euclidean distance), as shown by the dendrogram above. The colour bar at the top indicates sample group (blue: Ac only; orange: Ac/Kp). Each row represents one protein, labelled with its UniProt accession number and abbreviated protein description. The C-type lysozyme/α-lactalbumin superfamily protein (L8GZI4), identified as the priority candidate excretory–secretory protein (ESP), is highlighted in red bold text and marked with a star symbol. The colour scale (*z*-score of row-normalised log_2_ abundance) is shown to the right.

### Proteins with increased extracellular abundance in Ac/Kp co-culture

The 20 proteins showing the greatest increase in extracellular abundance in the Ac/Kp condition are listed in Table 1. Of these, 12 proteins were detected exclusively in the Ac/Kp conditioned medium (i.e. absent from all three Ac only replicates; log_2_ ratio = ∞). These included signal recognition particle docking protein (L8GKU9; 70.2 kDa), profilin (L8HBM2; 13.4 kDa), malate synthase (L8HCT0; 62.2 kDa), an aldo/keto reductase (L8GKL7; 36.3 kDa), actophorin (L8HMC3; 15.5 kDa), glucose-6-phosphate isomerase (L8GRA3; 63.3 kDa), an uncharacterised protein (L8GG92; 25.0 kDa), a mitochondrial intermembrane space Cx9C protein (L8GPR1; 19.6 kDa), an H^+^-transporting ATPase subunit (L8HH66; 64.5 kDa), phosphopyruvate hydratase (enolase; L8GRZ9; 46.6 kDa), inosine-uridine preferring nucleoside hydrolase (L8H1F8; 33.9 kDa), and a SnoaL-like domain protein (L8HJK2; 11.7 kDa). The remaining eight proteins showed quantitatively increased abundance, with log_2_ ratios ranging from +3.71 to +6.31 (Table 1).

**Table 1:** Top 20 proteins with the greatest increase in extracellular abundance in Ac/Kp conditioned medium. Proteins are ranked by decreasing extracellular abundance ratio (Ac/Kp *vs.* Ac only), with proteins detected exclusively in the Ac/Kp condition (log_2_ ratio = ∞) listed first, ranked by mean normalised abundance in Ac/Kp. Columns: Accession, UniProt accession number; Protein description, abbreviated functional annotation; MW (kDa), theoretical molecular weight; log_2_(ratio)^a^, log_2_-transformed abundance ratio (Ac/Kp/Ac only); *p*-value^b^, Welch’s two-sample *t*-test *p*-value; Detected Ac/Kp^c^, number of Ac/Kp replicates in which the protein was detected with “High” confidence. ∞, protein detected exclusively in the Ac/Kp condition (absent from all three Ac only replicates); –, *p*-value not calculable (protein absent from one group). The C-type lysozyme/α-lactalbumin superfamily protein (L8GZI4) is shown in bold as the priority candidate ESP.

Accession	Protein description	MW (kDa)	log_2_(ratio)^a^	*p*-valueᶜ	Detected Ac/Kp
L8GKU9	Signal recognition particle docking protein	70.2	∞	–	2/3
L8HBM2	Profilin	13.4	∞	–	2/3
L8HCT0	Malate synthase	62.2	∞	–	3/3
L8GKL7	Aldo/keto reductase family protein	36.3	∞	–	2/3
L8HMC3	Actophorin (putative)	15.5	∞	–	3/3
L8GRA3	Glucose-6-phosphate isomerase	63.3	∞	–	2/3
L8GG92	Uncharacterized protein	25.0	∞	–	3/3
L8GPR1	Mitochondrial IMS Cx9C protein	19.6	∞	–	2/3
L8HH66	H^+^-transporting two-sector ATPase subunit	64.5	∞	–	2/3
L8GRZ9	Phosphopyruvate hydratase (enolase)	46.6	∞	–	2/3
L8H1F8	Inosine-uridine preferring nucleoside hydrolase	33.9	∞	–	2/3
L8HJK2	SnoaL-like domain protein	11.7	∞	–	2/3
L8GPA7	EF-hand domain-containing protein	16.9	+6.31	0.0083	3/3
L8GM55	RNA-binding motif protein	47.5	+5.68	0.0117	3/3
L8GG56	High molecular weight heat shock protein	72.2	+5.57	0.0128	3/3
L8HKB3	Cytochrome c (putative)	13.0	+4.00	0.0289	3/3
L8H1F5	CS domain-containing protein	22.4	+3.78	0.0351	3/3
L8HKD4	Calcium-regulated actin-bundling protein	64.5	+3.75	0.0362	3/3
L8H373	14-3-3 domain-containing protein	36.3	+3.71	0.0381	3/3
**L8GZI4**	**C-type lysozyme/α-lactalbumin superfamily proteinᵇ**	**14.7**	**+5.19**	**0.0168**	**3/3**

^a^log_2_(ratio): log_2_-transformed abundance ratio (Ac/Kp / Ac only). ∞, protein detected exclusively in the Ac/Kp condition (absent from all three Ac only replicates). ^b^*p*-value: Welch’s two-sample t-test. —, *p*-value not calculable (protein absent from one group). ^c^Detected Ac/Kp: number of Ac/Kp replicates in which the protein was detected with “High” confidence. ᵇ Bold italic: priority candidate excretory–secretory protein.

### Identification of C-type lysozyme as a primary candidate protein

Among all proteins with increased extracellular abundance, the C-type lysozyme/α-lactalbumin superfamily protein (UniProt accession L8GZI4; 14.7 kDa) was identified as the priority candidate, as it is the only protein in the increased-abundance set that fulfils three convergent criteria: (i) classical secreted protein family with well-established extracellular function; (ii) molecular weight consistent with the active excretory–secretory protein (ESP) fraction previously identified in our laboratory as capable of inducing *K. pneumoniae* capsule enlargement; and (iii) mechanistic product compatibility with the Rcs two-component system. L8GZI4 exhibited a log_2_(ratio) of +5.19 (*p* = 0.0168) and was detected in all three Ac/Kp replicates, but only one Ac only replicate, indicating a marked increase in extracellular release during co-culture (Table 1). As a member of the C-type lysozyme superfamily, this protein catalyses the hydrolysis of β-1,4-glycosidic bonds in bacterial peptidoglycan, generating muropeptides with molecular masses typically below 2 kDa. These muropeptide products fall well within the active <10 kDa fraction previously shown to activate the Rcs phosphorelay and upregulate downstream capsule biosynthesis genes, including *rcsB*, *galF*, and *wzi*, in *K. pneumoniae*. Of note, owing to the well-documented detection bias of bottom-up LC-MS/MS proteomics against low-molecular-weight proteins, no proteins with a molecular mass below 10 kDa were identified in the present study, precluding direct proteomic characterisation of the active ESP fraction.

### Functional enrichment analysis of increased-abundance proteins

Gene Ontology (GO) functional category enrichment analysis was performed on the 28 *A. castellanii* proteins with increased extracellular abundance in the Ac/Kp condition (log_2_ ratio > 0, detected in ≥ 2 of 3 Ac/Kp replicates) against the background of all 391 identified proteins. Fisher’s exact test identified five significantly enriched functional categories (*p* < 0.05; Fig. 5A): Transport (*n* = 4 proteins; *p* < 0.001), Signaling/Regulation (*n* = 4; *p* = 0.0049), Redox/Electron Transport (*n* = 4; *p* = 0.0113), Ion/Metal Binding (*n* = 3; *p* = 0.0167), and Chaperone/Stress Response (*n* = 2; *p* = 0.0295). The Antimicrobial/Defense category, containing the C-type lysozyme candidate (L8GZI4), showed a trend towards enrichment (*n* = 1; *p* = 0.0580). These enrichment patterns collectively suggest that co-culture with *K. pneumoniae* alters not only the quantity but also the functional composition of the *A. castellanii* extracellular proteome, with a shift towards stress-responsive, redox-active, and signalling-related proteins.

**Figure 5:**
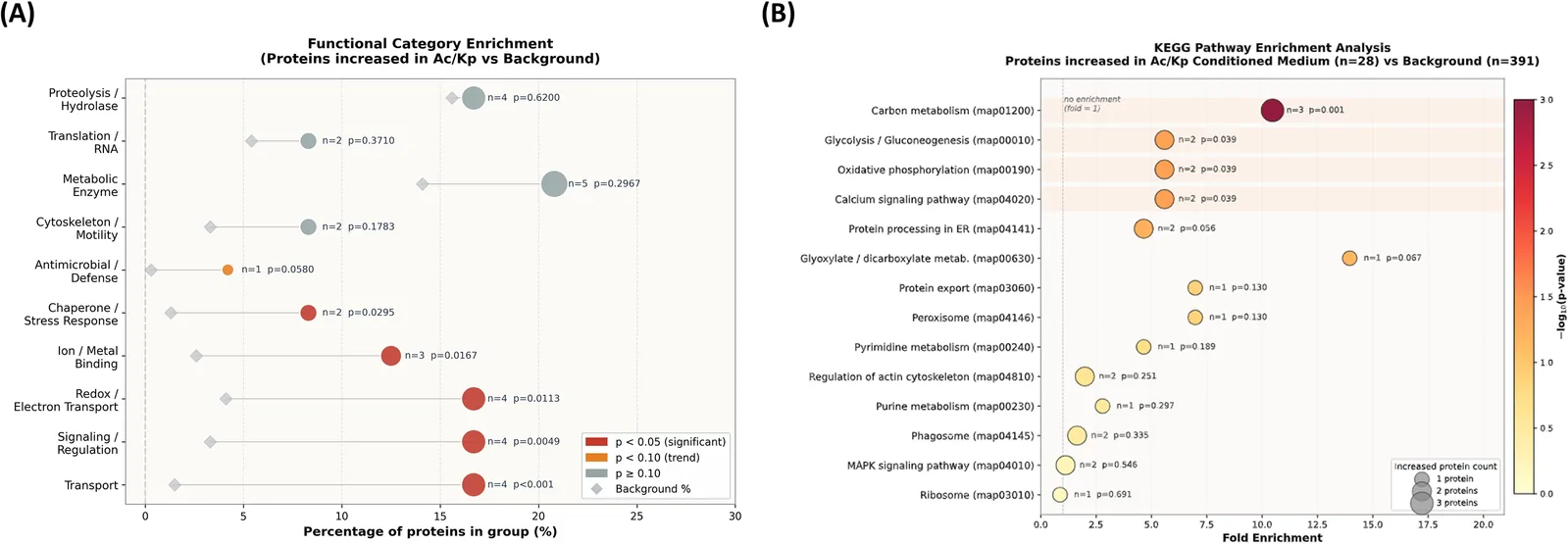
Functional enrichment analyses of proteins with increased extracellular abundance in Ac/Kp conditioned medium. Enrichment analyses were performed on the set of 28 *A. castellanii* proteins with increased extracellular abundance in the Ac/Kp condition (log_2_ ratio > 0; detected in ≥2 of 3 Ac/Kp replicates), using all 391 identified proteins as the background reference. Significance was assessed by Fisher’s exact test (one-sided). (A) Gene Ontology (GO) functional category enrichment. Enriched categories are plotted as a lollipop chart; filled circles indicate significance level (orange: *p* < 0.05; blue: *p* < 0.10 trend; grey: *p* ≥ 0.10). Circle size is proportional to the percentage of foreground proteins in each category. The background percentage for each category is shown as a grey diamond. (B) KEGG pathway enrichment analysis. Enriched pathways are plotted as a bubble chart; bubble size is proportional to the number of foreground proteins contributing to each pathway, and bubble colour represents the negative log_10_-transformed *p*-value (colour scale shown at right). The vertical dashed line indicates fold enrichment = 2.

### KEGG pathway enrichment analysis

KEGG pathway enrichment analysis of the same increased-abundance protein set identified four significantly enriched pathways (*p* < 0.05; Fig. 5B). Carbon metabolism (map01200) showed the greatest enrichment (*n* = 3 proteins; fold enrichment ≈ 10; *p* = 0.001), contributed by malate synthase (L8HCT0), phosphopyruvate hydratase (L8GRZ9), and glucose-6-phosphate isomerase (L8GRA3), all of which were detected exclusively in the Ac/Kp condition. Glycolysis/Gluconeogenesis (map00010; *n* = 2; *p* = 0.039), Oxidative phosphorylation (map00190; *n* = 2; *p* = 0.039), and the Calcium signalling pathway (map04020; *n* = 2; *p* = 0.039) were also significantly enriched. A trend towards enrichment was observed for Protein processing in the endoplasmic reticulum (map04141; *n* = 2; *p* = 0.056). The enrichment of carbon metabolism and energy-related pathways may reflect an increased metabolic demand or altered energy channelling in *A. castellanii* during active interaction with *K. pneumoniae*.

## Discussion

Free-living amoebae such as *Acanthamoeba* spp. are recognised as environmental training grounds for bacterial pathogens, where predator–prey interactions select for virulence traits that subsequently enhance pathogenicity in mammalian hosts [21]. Environmental amoebae, including species closely related to *A. castellanii* (AcT4), actively predate on *Klebsiella pneumoniae* in natural settings, and the bacterium’s capsular polysaccharide has been shown to modify, though not prevent, amoebal predation kinetics [1]. Maisonneuve *et al.* demonstrated specifically that *K. pneumoniae* growth is enhanced during co-culture with *A. castellanii*, and that amoeba-conditioned medium promotes bacterial proliferation [16], suggesting that co-culture with *Klebsiella* activates bidirectional molecular responses in *A. castellanii*. The present study provides the first proteomic characterisation of the AcT4 extracellular response to *K. pneumoniae* encounter. Mass spectrometry-based secretomics of conditioned medium represents an unbiased approach to cataloguing extracellular proteins, though it is inherently challenged by the co-presence of classically and unconventionally secreted proteins and by the well-documented detection bias against proteins below approximately 10 kDa in bottom-up tryptic workflows [12]. The 391 *A. castellanii* proteins identified in our study span a broad functional landscape, and the bidirectional remodelling of the conditioned medium proteome (with 25 proteins appearing exclusively under co-culture conditions and 138 reduced or absent) is consistent with published observations in the *A. castellanii* secretome field. Comparative proteomic analyses of *A. castellanii* extracellular proteins have previously shown condition-specific gains and losses of secreted proteins, including protease enrichment in pathogenic *versus* non-pathogenic strains [10, 19] and selective enrichment of glycolytic enzymes, oxidative metabolism proteins, and carbohydrate hydrolases under nutritional stress [6]. The change in Ac extracellular protease profile after co-culture with mammalian cells further supports the view that *Acanthamoeba* systematically adjusts its secretory output in response to living target organisms [5]. The GO and KEGG enrichment patterns we observed in the Ac/Kp increased-abundance proteins, encompassing Carbon metabolism, Transport, Redox, Ion/Metal Binding, Chaperone/Stress Response, and Calcium signalling, extend these published secretome patterns into the context of protozoa–bacteria co-culture, and parallel the shifts documented for *A. castellanii* under environmental stress.

Among the proteins with significantly increased extracellular abundance in Ac/Kp conditioned medium, the C-type lysozyme/α-lactalbumin superfamily protein (L8GZI4; 14.7 kDa; log_2_ ratio = +5.19; *p* = 0.0168) is identified as the priority candidate excretory–secretory protein (ESP) based on three converging lines of evidence. First, C-type lysozymes are canonical ~14 kDa secreted antimicrobial enzymes that catalyse hydrolysis of the β-1,4 glycosidic bond between N-acetylmuramic acid and N-acetylglucosamine in bacterial peptidoglycan, generating muropeptide fragments typically below 2 kDa [22]. This enzymatic function is entirely consistent with the primary predatory secretory repertoire of *A. castellanii*: in a manner analogous to the secreted aminopeptidase that disrupts target cells to facilitate phagocytosis [9], C-type lysozyme may represent an additional enzymatic weapon deployed during bacterial predation, with its hydrolysis products incidentally serving as inter-kingdom signalling molecules. Second, the mechanistic link between C-type lysozyme activity and Rcs phosphorelay activation in Enterobacteriaceae is experimentally established: direct exposure of *Escherichia coli* to C-type lysozyme activates the Rcs regulon through the outer membrane lipoprotein sensor RcsF [2], while the Rcs system more broadly responds to any perturbation of the peptidoglycan layer [13]. Elevated periplasmic muropeptide concentrations generated by enzymatic hydrolysis have been proposed as the specific molecular cues detected by RcsF, initiating downstream phosphotransfer through RcsC–RcsD–RcsB [14]. In *K. pneumoniae* specifically, RcsB and the RcsAB heterodimer govern the expression of the *cps* gene cluster responsible for capsular polysaccharide biosynthesis, and the Rcs system is regarded as a conserved regulator of capsule synthesis in response to envelope stress across *Enterobacteriaceae* [17]. Third, the broader secretome enrichment of Ion/Metal Binding proteins (*p* = 0.0167) is noteworthy given that iron homeostasis and capsule regulation are interconnected in *K. pneumoniae*: RcsAB and the ferric uptake regulator Fur co-regulate the iron acquisition gene *entC* in a manner linking iron depletion to Rcs-dependent gene activation [30], and the iron–sulfur cluster regulator IscR directly enhances capsular polysaccharide biosynthesis gene expression in response to iron and oxidative stress [29]. Notably, the hypermucoviscous capsule phenotype that arises from enhanced *cps* expression is a defining feature of hypervirulent *K. pneumoniae* (hvKp) [18, 27], which is a growing global public health concern increasingly detected in freshwater aquatic environments [28]. Our findings suggest that AcT4 co-culture creates conditions in which C-type lysozyme-derived muropeptides activate the Rcs phosphorelay, while amoeba-secreted ion/metal-binding proteins may further modulate iron-regulatory networks. Together, these signals may synergistically drive capsule upregulation and the hvKp-associated phenotype in *K. pneumoniae*.

Several limitations must be acknowledged. The bottom-up LC-MS/MS approach used here systematically underdetects proteins below approximately 10 kDa, because tryptic digestion of small proteins generates insufficient peptides for confident identification [3, 12]. Consequently, the active <10 kDa ESP fraction identified in our previous study as responsible for Rcs activation is not directly accessible by the present method; C-type lysozyme (14.7 kDa) was detectable precisely because it sits slightly above this threshold, but its mechanistically active muropeptide products (<2 kDa) were not detectable by this approach. Furthermore, only the *A. castellanii* proteome was used for database searching, so any *K. pneumoniae*-derived proteins present in the co-culture conditioned medium were not identified. Critically, no wet-laboratory validation of L8GZI4 was performed, and its candidate status rests entirely on proteomic evidence and mechanistic plausibility. Future work should prioritise (i) recombinant expression of L8GZI4 and direct testing of its enzymatic products on *K. pneumoniae* using an Rcs-responsive reporter system, (ii) fractionation of conditioned medium below 10 kDa followed by peptidomics or top-down mass spectrometry to directly characterise the active ESP fraction, (iii) determination of whether L8GZI4 is delivered as a soluble protein or via extracellular vesicles, given the documented functional activity of *Acanthamoeba*-derived EVs [25], and (iv) ecological validation in model freshwater systems combining both organisms at environmental densities.

## Conclusions

This study presents the first comparative secretome analysis of *A. castellanii* genotype T4 during co-culture with *K. pneumoniae*. Among 391 identified amoeba-derived extracellular proteins, the C-type lysozyme/α-lactalbumin superfamily protein (L8GZI4; 14.7 kDa; log_2_ ratio = +5.19) is identified as the priority candidate ESP, supported by the established connection between C-type lysozyme activity and Rcs phosphorelay activation, and by its mechanistic compatibility with the active <10 kDa ESP fraction and Rcs-dependent capsule gene upregulation documented in our previous studies. The broader secretome changes, including enrichment of carbon metabolic enzymes, redox-active proteins, and ion/metal-binding proteins, parallel stress-induced secretome shifts reported in other *A. castellanii* contexts and may synergise with Rcs-linked iron-regulatory circuits in *K. pneumoniae*. Taken together, these findings support a model in which *A. castellanii* functions not merely as a passive host or predator of *K. pneumoniae* but as an active modulator of bacterial virulence through secreted enzymatic signals, providing a molecular foundation for the amoeba-as-training-ground concept and offering novel targets for future investigation of environmentally driven hvKp emergence.
